# Multiple Modes of Nematode Control by Volatiles of *Pseudomonas putida* 1A00316 from Antarctic Soil against *Meloidogyne incognita*

**DOI:** 10.3389/fmicb.2018.00253

**Published:** 2018-02-23

**Authors:** Yile Zhai, Zongze Shao, Minmin Cai, Longyu Zheng, Guangyu Li, Dian Huang, Wanli Cheng, Linda S. Thomashow, David M. Weller, Ziniu Yu, Jibin Zhang

**Affiliations:** ^1^State Key Laboratory of Agricultural Microbiology and National Engineering Research Center of Microbe Pesticides, College of Life Science and Technology, Huazhong Agricultural University, Wuhan, China; ^2^Key Laboratory of Marine Biogenetic Resources, Third Institute of Oceanography, State Oceanic Administration, Xiamen, China; ^3^Wheat Health, Genetics and Quality Research Unit, Agricultural Research Service, United States Department of Agriculture, Pullman, WA, United States

**Keywords:** *Pseudomonas putida* 1A00316, *Meloidogyne incognita*, chemotaxis, egg hatching, volatile organic compound, nematode control

## Abstract

*Pseudomonas putida* 1A00316 isolated from Antarctic soil showed nematicidal potential for biological control of *Meloidogyne incognita*; however, little was known about whether strain 1A00316 could produce volatile organic compounds (VOCs), and if they had potential for use in biological control against *M. incognita*. In this study, VOCs produced by a culture filtrate of *P. putida* 1A00316 were evaluated by *in vitro* experiments in three-compartment Petri dishes and 96-well culture plates. Our results showed that *M. incognita* juveniles gradually reduced their movement within 24–48 h of incubation with mortality ranging from 6.49 to 86.19%, and mostly stopped action after 72 h. Moreover, egg hatching in culture filtrates of strain 1A00316 was much reduced compared to that in sterile distilled water or culture medium. Volatiles from *P. putida* 1A00316 analysis carried out by solid-phase micro-extraction gas chromatography–mass spectrometry (SPME-GC/MS) included dimethyl-disulfide, 1-undecene, 2-nonanone, 2-octanone, (Z)-hexen-1-ol acetate, 2-undecanone, and 1-(ethenyloxy)-octadecane. Of these, dimethyl-disulfide, 2-nonanone, 2-octanone, (Z)-hexen-1-ol acetate, and 2-undecanone had strong nematicidal activity against *M. incognita* J2 larvae by direct-contact in 96-well culture plates, and only 2-undecanone acted as a fumigant. In addition, the seven VOCs inhibited egg hatching of *M. incognita* both by direct-contact and by fumigation. All of the seven VOCs repelled *M. incognita* J2 juveniles in 2% water agar Petri plates. These results show that VOCs from strain 1A00316 act on different stages in the development of *M. incognita* via nematicidal, fumigant, and repellent activities and have potential for development as agents with multiple modes of control of root-knot nematodes.

## Introduction

Plant parasitic nematodes (PPNs) cause serious damage to a wide range of crops worldwide (Li et al., 2015). To date, more than 4100 species of PPNs have been described (Kyndt et al., 2014). Among these, the root-knot nematode (*Meloidogyne* spp.), found all over the world in tropical, subtropical, and temperate regions, is the most economically important plant nematode (Jones et al., 2013). The destruction caused by PPNs has been assessed to be more than 100–150 billion US dollars per year, of which more than half are due to *Meloidogyne* spp. (Nicol et al., 2011; Li et al., 2015; Kim et al., 2016). Among the *Meloidogyne* spp., *Meloidogyne incognita* is the most destructive because of its wide host range, including most flowering plants, as well as its short generation time, high reproduction rate, and ability to form complex diseases with other soil-borne pathogens such as fungi (Vos et al., 2013). *M. incognita* can infect the roots of over 2000 plant species and interferes with normal plant uptake of nutrients and water. Moreover, it causes physiological plant disorders (Jang et al., 2014), making it perhaps the most damaging of all crop pathogens (Trudgill and Blok, 2001). At present, the main method of controlling plant nematodes involves using chemical nematicides. However, these chemicals are toxic, have side effects against other organisms, and can adversely affect human health and the environment (Riga, 2011). The rotation and resistance of crop varieties are complementary strategies to control PPNs, but their effectiveness is limited (Jones et al., 2013). Therefore, new economical, effective, and environmentally friendly PPN controls are urgently needed.

Biological control is one such way to reduce pest losses, but more investigation on novel microorganisms in the environment is needed to speed the development of new agents for controlling root-knot nematodes (Li et al., 2016). These pathogens inhabit the soil and typically are infected with indigenous bacteria and fungi, suggesting the possibility of using microorganisms to control PPNs (Zhang et al., 2015). In fact, there are specific microorganisms in soil, such as nematophagous bacteria and fungi, which have complex strategies for capturing, killing, and digesting PPNs, and they often target a specific stage of the nematode life cycle (Li et al., 2015). Many secondary metabolites of microorganisms have nematicidal activity (NA) and therefore could become substitutes for highly toxic chemical nematicides. Through comparative genomic studies, isolation and purification, the nematicidal compounds alkaline metalloproteinase AprA and two metabolites, hydrogen cyanide and cyclo(L-Pro-L-Ile), were previously identified from *Pseudomonas putida* strain 1A00316 and shown to have good NA against *M. incognita* (Guo et al., 2016). *Bacillus cereus* strain S2 produced sphingosine and showed high NA against *M. incognita* (Gao et al., 2016). Moreover, *Purpureocillium lilacinum*, with the adjuvant avermectin, has been used effectively to control PPNs (Fisher, 1990; Kiewnick and Sikora, 2006).

*Pseudomonas* species are ubiquitous in nature and produce many secondary metabolites active against important plant pathogens (Giles et al., 2014). Members of the genus are physiologically and metabolically multifunctional, readily colonizing terrestrial and aquatic habitats such as soil, plants, and water (Troxler et al., 2012). Among the species of this genus, *P. putida* has been isolated from many niches and survives in soil containing organic pollutants and heavy metals. For example, *P. putida* JMQS1 isolated from detergent-contaminated soil exhibited quorum sensing along with its ability to degrade phenol (Antony and Jayachandran, 2016). Some strains of *P. putida* inhabit the rhizosphere or are endophytes that promote plant growth, making them ideal for biocontrol. The nematicidal effects of *P. putida* against *M. incognita* were noted previously; strain 1A00316 isolated from Antarctic soil showed good inhibition of *M. incognita in vitro* and in pot experiments, with biocontrol efficiency of nematodes as high as 71.67%. In addition, strain 1A00316 itself could induce systematic resistance in tomato by increasing the activity of three defense enzymes: phenylalanine ammonia lyase, polyphenol oxidase, and peroxidase in tomato plants (Tang et al., 2014). Hydrogen cyanide and cyclo(L-Pro-L-Ile) also were identified from strain 1A00316 and exhibited NA against *M. incognita* (Guo et al., 2016), but little is known about whether volatile organic compounds (VOCs) are produced by the strain, or if they have potential for use in biological control against *M. incognita.*

Compared to solid nematicides, the greatest advantages of volatile nematicidal compounds are their good dispersibility and penetration in the soil. In the past, fumigants such as methyl bromide, which was used as nematicide, have been identified as contributing to the reduction of the ozone layer and overall poor air quality, so it is important to search for new green soil fumigation agents. Accordingly, this investigation focused on the identification and evaluation of VOCs from strain 1A00316. In the present study, we report (i) the identification the volatile compounds in the fermentation broth of *P. putida* 1A00316; and (ii) evaluation of their nematicidal, fumigation, and chemotaxis activities against *M. incognita.*

## Materials and Methods

### Chemical Compounds

Dimethyl-disulfide and 2-nonanone were purchased from TCI (Shanghai, China) with a purity >98%. 1-undecene (>99.5%), 2-octanone (>99%), (Z)-hexen-1-ol acetate (99%), and 1-(ethenyloxy)-octadecane (90%) were purchased from Yuan Ye (Shanghai, China). 2-undecanone was purchased from Sigma Aldrich (Shanghai, China) with a purity >99%. Methanol, Tween-20 and activated charcoal were purchased from Sinopharm Chemical Reagent Company (Shanghai, China).

### Collection of *M. incognita* Eggs and Second-Stage Juveniles and Propagation of *Caenorhabditis elegans*

*Meloidogyne incognita* eggs were collected from the roots of infested tomato plants (*Solanum lycopersicum* L.), which were previously infected with the nematodes in the greenhouse at 23–26°C, and relative humidity 40–60%. The tomato plants were watered manually once a day. After 45 days, the plants were uprooted, the roots were rinsed free of soil with tap water, and the egg masses were picked into a bottle with a dissecting needle (Lee et al., 2014). After shaking the egg mass with 1% NaOCl (sodium hypochlorite) solution by hand in the bottle for 3 min, the solution was passed in turn through a series of filters with pore sizes of 74, 45, and 25 μm, and the sterilized eggs were collected from the 25-μm filter by spraying with sterile distilled water (SDW; Seo et al., 2013). Second-stage juveniles of *M. incognita* were obtained by using a modified Baermann funnel method under sterile conditions (Barker et al., 1985; Southey, 1986). *Caenorhabditis elegans* N2 (Bristol, wild type), was purchased from the Caenorhabditis Genetics Center (CGC). *C. elegans* was maintained at 20°C on nematode growth medium plates seeded with *Escherichia coli* OP50.

### Preparation of Fermentation Broth of Strain *P. putida* 1A00316

Strain 1A00316 was isolated from Antarctic soil and identified as *P. putida* by sequence homology of the 16S rDNA and physiological and biochemical characteristics (Tang et al., 2014). The strain was cultured in 30-mL flasks containing 15 mL of 2216E broth (Morisaki et al., 1999) prepared from 10 g peptone, 5 g yeast powder, 1 g beef extract, 0.1 g ferric citrate, 1 g sodium acetate, 19.45 g NaCl, 0.75 g MgCl_2_, 0.75 g MgSO_4_, 1 g CaCl_2_, 0.55 g KCl, 0.16 g NaHCO_3_, 0.08 g KBr, 34 mg SrCl_2_, 22 mg H_3_BO_3_, 4 mg Na_2_SiO_3_, 2.4 mg NaF, 8 mg Na_2_HPO_4_, 0.5 mg MnCl_2_, 0.5 mg CuSO_4_, and 10 mg ZnSO_4_ and adjusted to a pH value of 7.6–7.8. A seed culture was incubated at 28°C, shaken at 180 rpm, and 1% seed liquid (2.5 mL) was transferred after 18 h, to 500-mL flasks containing 250 mL of 2216E medium. The cultures were shaken as above for 48 h and then centrifuged at 4225 × *g* for 10 min at 4°C to obtain the supernatant, which was passed through a 0.22-μm filter to remove bacterial cells. The filtrates were used at the original concentration and diluted 1/3, 1/5, 1/10, 1/15, and 1/20 with SDW.

### Nematicidal Activity of Volatiles

**FIGURE 1:**
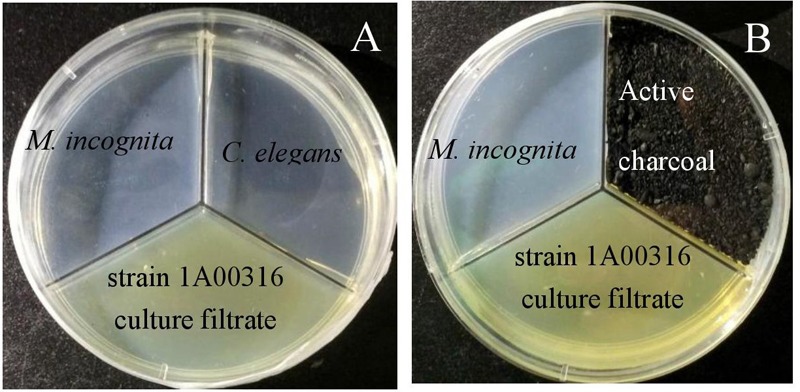
Nematicidal activity of *P. putida* strain 1A00316 in Petri plates **(A,B)**.

A three-compartment Petri plate (**Figure 1A**; 85 mm diameter; Fernando et al., 2005) was used to study the NA of bacterial VOCs. Three milliliters of the original culture filtrate was added into one compartment and 200 nematodes of either *M. incognita* or *C. elegans* were introduced onto the surface of layers of 2% water agar (WA) in the other two compartments. Control plates contained uninoculated 2216E medium in place of the culture filtrate. Plate lids were immediately sealed with Parafilm (Bemis) to avoid escape of the volatiles, and the plates were incubated at 28°C in the dark. There were three replicates for each treatment and the experiments were repeated twice. After 24, 48, and 72 h, the numbers of mobile and immobile nematodes were counted under a dissecting microscope (Jiang Nan JS25B).

In order to confirm the NA of bacterial VOCs produced by strain 1A00316, activated charcoal was introduced into one of the three compartments and culture supernatant and nematodes were added to each of the other two compartments of the Petri plate (**Figure 1B**; Gu et al., 2007). The activated charcoal can adsorb volatiles, blocking their activity and resulting in no loss of nematode viability. At the same time, 100 eggs of *M. incognita* were immersed in 100 μL of SDW in wells of 96-well tissue culture plates surrounded by four adjacent wells containing culture filtrate of strain 1A00316. SDW and 2216E media in the surrounding wells served as controls. The experiment was repeated three times. The numbers of eggs hatching were counted after 2, 4, 6, 8, and 10 days of exposure with an inverted microscope (XDS-1B COIC, Chongqing Mike Photoelectric Instrument Limited Company, China).

### Identification of Volatiles from Strain 1A00316

Strain 1A00316 was cultured for 48 h as described above, and volatiles were collected and analyzed by using SPME-GC/MS (Azenha and Vasconcelos, 2002; Dìaz et al., 2004). Fiber (65 μm PDMS/DVB fiber, Supelco, Bellefonte, PA, United States) used for SPME was first preconditioned with helium at 250°C for 20 min. The extractions were performed in 20-mL headspace vials (22.5 mm × 75.5 mm) filled with 9 mL fermentation broth and a magnetic stirring bar. The vials were fixed inside a thermostatic water bath and samples were equilibrated at 60°C for 1 h. The VOCs from 9 mL 2216E medium were used as controls. After the extraction, the fiber was inserted into the injection port of a gas chromatograph [Hewlett-Packard (HP) 7890A] coupled with a mass spectrometer (HP 5975C, Agilent Technologies, United States) and desorbed for 5 min at 250°C (Gu et al., 2007). The chromatographic separation was performed on a HP-5MS (30 m × 0.25 mm) × 0.25 μL column and helium was used as the carrier gas at a constant flow of 1 mL/min. The column was held at 40°C for 2 min, then increased to 180°C at a rate of 4°C/min, held for 0 min, then increased to 240°C at a rate of 5°C/min, and held for 6 min. The MS detector was programmed as follows: EI ion source operating at 70 eV, acquisition range between *m/z* 35 and 550. The temperature of the transfer line and iron trap were 250 and 300°C, respectively. The identification of a volatile compound was based on a comparison of the substance with GC/MS system data banks (NIST 08 Library). Each sample was tested twice.

### Nematicidal Activity of Commercial VOCs

The NA of commercial VOCs was tested against *M. incognita* J2 larvae at a dose range of 10–1000 mg/L, and the 50% lethal concentration (LC_50_) values were calculated. Stock solutions of pure compounds were prepared in methanol to overcome insolubility, whereas aqueous Tween-20 (0.3% v/v) was used for further dilution. Test solutions (200 μL) at various concentrations were added to detachable 96-well tissue culture plates and a suspension of 40–50 J2 juveniles was added to each well. SDW and a mixture of methanol and Tween-20 served as controls (Aissani et al., 2013). Plate lids were sealed with Parafilm to avoid evaporation and plates were kept at 20°C in the dark. Juveniles in solutions of VOCS were observed with the aid of an inverted microscope after 24, 48, and 72 h and were categorized as either motile or immotile/paralyzed. The experiments were performed three times, and every treatment was replicated three times.

### Fumigant Activity of VOCs against J2

The various commercial VOCs were introduced into one well in 96-well tissue culture plates surrounded by four wells containing 50 nematode J2 juveniles suspended in distilled water. SDW and a mixture of methanol and Tween-20 served as controls. Percentages of nematode death were recorded in response to the fumigant activity of the VOCs in the adjacent wells. Assessments were made at 24, 48, and 72 h (Ntalli et al., 2011). The experiments were performed three times, and every treatment was replicated three times.

### Effect of VOCs on Egg Hatching

Whether VOCs could inhibit egg hatching of *M. incognita* was tested over a dose range of 20–1000 mg/L. One hundred eggs of *M. incognita* suspended in 10 μL SDW were introduced into detachable 96-well tissue culture plates and combined with 200 μL of a range of concentrations of commercial VOCs. SDW as well as a mixture of methanol and Tween-20 served as controls. Each treatment had three replicates and the experiments were repeated twice.

At the same time, 100 eggs of *M. incognita* in 100 μL SDW were introduced into wells of 96-well tissue culture plates and surrounded by four wells containing 200 μL of one of the VOCS at 1000 μg/mL. SDW and 2216E media served as controls. Plate lids were sealed with Parafilm and plates were incubated in the dark at 20°C. The numbers of J2 hatchings were counted after 2, 4, 6, 8, and 10 days of exposure under the inverted microscope and hatch rate was measured. Each treatment had four replicates and the experiments were repeated twice.

### Chemotaxis of J2 Nematodes to Culture Filtrate and VOCs

**FIGURE 2:**
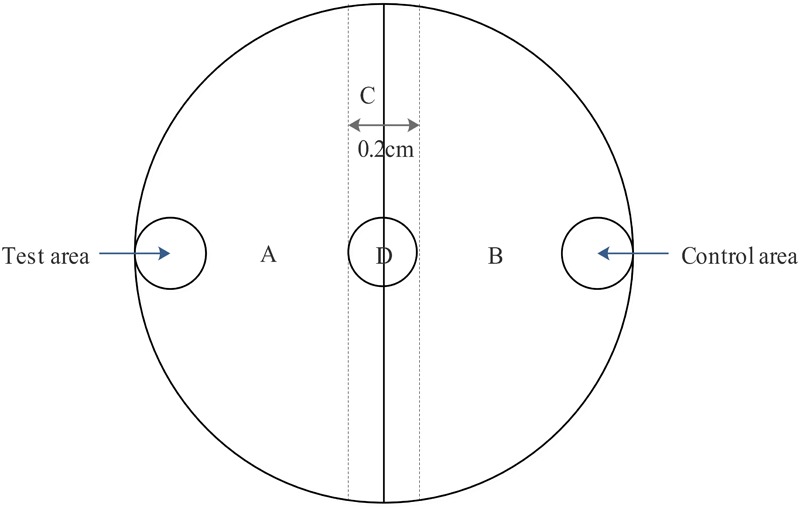
Configuration of the *M. incognita* chemotaxis assay plate.

Chemotaxis was assessed on Petri plates containing 2% WA (Tajima et al., 2001). A 5 mm filter paper disc immersed in various concentrations of culture filtrate or solutions of volatile substances was placed on the test area (A) of 35 mm Petri dishes, while a filter paper immersed in 2216E medium or a mixture of methanol and Tween-20 was added to the opposite side of the plate (area B) as a control (**Figure 2**). Subsequently, 150 J2 juveniles of *M. incognita* were added to the center (area D) of the Petri dish, and the dish was incubated in a dark cabinet at 20°C for 8 h (Hu et al., 2012). The numbers of J2s in areas A and B were then counted under a dissecting microscope to calculate the chemotaxis index (C.I.; Saeki et al., 2001), calculated after 8 h as.

C.I. = (the number of nematodes in test area - the number of nematodes in control area)/(the number of nematodes in test area + the number of nematodes in control area).

For 0 < C.I. < 1, *M. incognita* was attracted to the tested sample; if -1 < C.I. < 0, the tested sample repelled *M. incognita*; and if C.I. = 0, the sample had no effect on the nematode. Experiments were performed in triplicate, and treatments were replicated three times.

### Data Analysis and Statistics

Mortality values for *in vitro* bioassays against *M. incognita* were corrected by Abbott’s formula (Abbott, 1925). The LC_50_ was calculated by Probit analysis. Data from the chemotaxis assay were analyzed using a homogeneity test of variance. If the variance was homogeneous (*P* ≤ 0.05), a paired Student’s *t*-test was chosen; otherwise, the Wilcoxon rank sum test was used. Data from all assays except the chemotaxis assay were analyzed by one-way variance with SPSS 20. Means among treatments were compared by Fisher’s least significant difference (LSD) test at the *P* = 0.05 level.

## Results

### Nematicidal Effects of Strain 1A00316 VOCs

**FIGURE 3:**
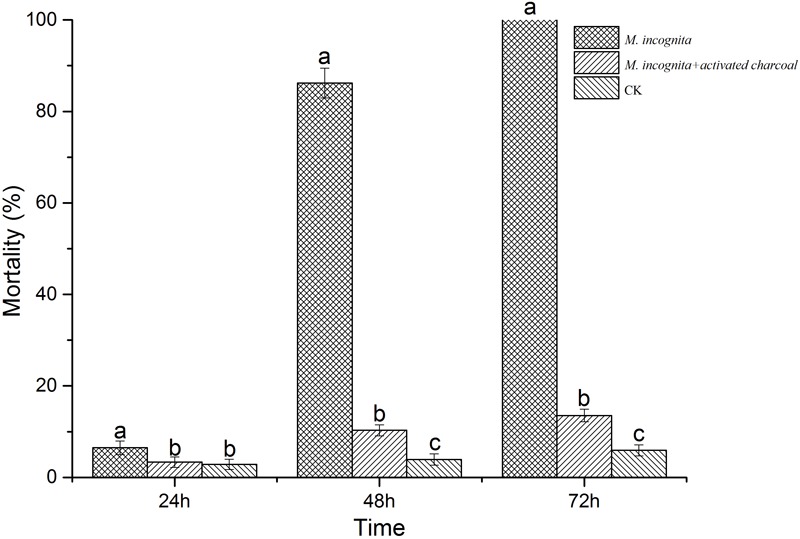
Nematicidal effects of strain 1A00316 VOCs against *M. incognita* in a three-compartment Petri plate. Values with the same lowercase letters do not differ from each other at *P* < 0.05; bars indicate the standard error of the means (*n* = 3).

**FIGURE 4:**
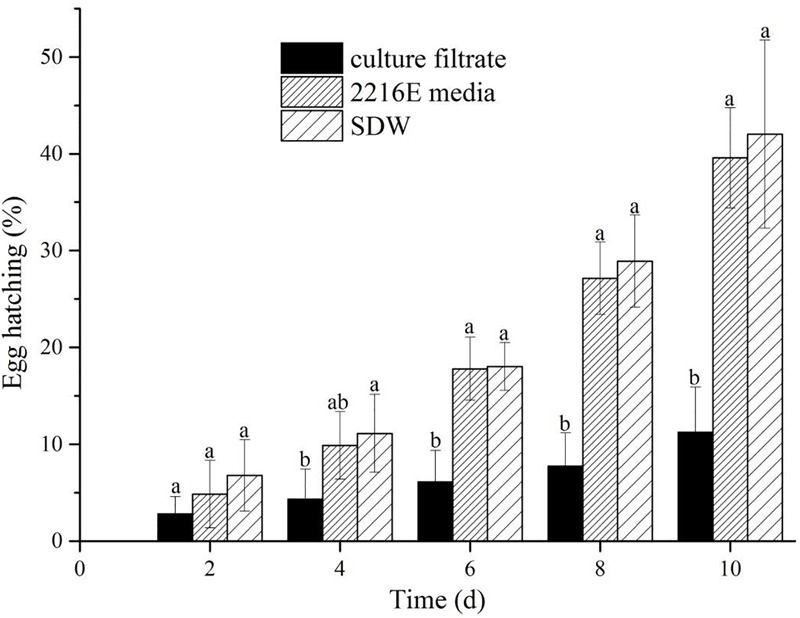
Fumigation effects of original culture filtrates on egg hatching of *M. incognita* after 2, 4, 6, 8, and 10 days of incubation. Values with the same lowercase letters do not differ from each other at *P* < 0.05; bars indicate the standard error of the means (*n* = 4).

We evaluated effects of VOCs produced by culture filtrate on *M. incognita* J2s by the three-compartment Petri plate method. Juveniles of *M. incognita* gradually reduced movement within 24–48 h and mostly were immobile after 72 h of incubation, with mortality ranging from 6 to 100% (**Figure 3**). We also found that strain 1A00316 had strong NA (≥80%) against *M. incognita* J2 juveniles, but not to *C. elegans* (Supplementary Table S1), indicating that *C. elegans* was not sensitive to these VOCs. Moreover, most of the VOCs were adsorbed in the plates containing activated charcoal, and NA values decreased from 86% to less than 10%. In addition, neither culture filtrate nor 2216E medium affected egg hatching after 2 and 4 days, although hatching slowly declined after 6 days, and reached 11.24% at 10 days, a value significantly less than control values in SDW or 2216E medium (**Figure 4**). These results suggest that the culture filtrate of strain 1A00316 contains VOCs that kill nematodes and inhibit egg hatching, and they are consistent with the hypothesis that VOCs from strain 1A00316 are responsible for the NA.

### Nematicidal Activity of Commercial VOCs against J2 Nematodes

**FIGURE 5:**
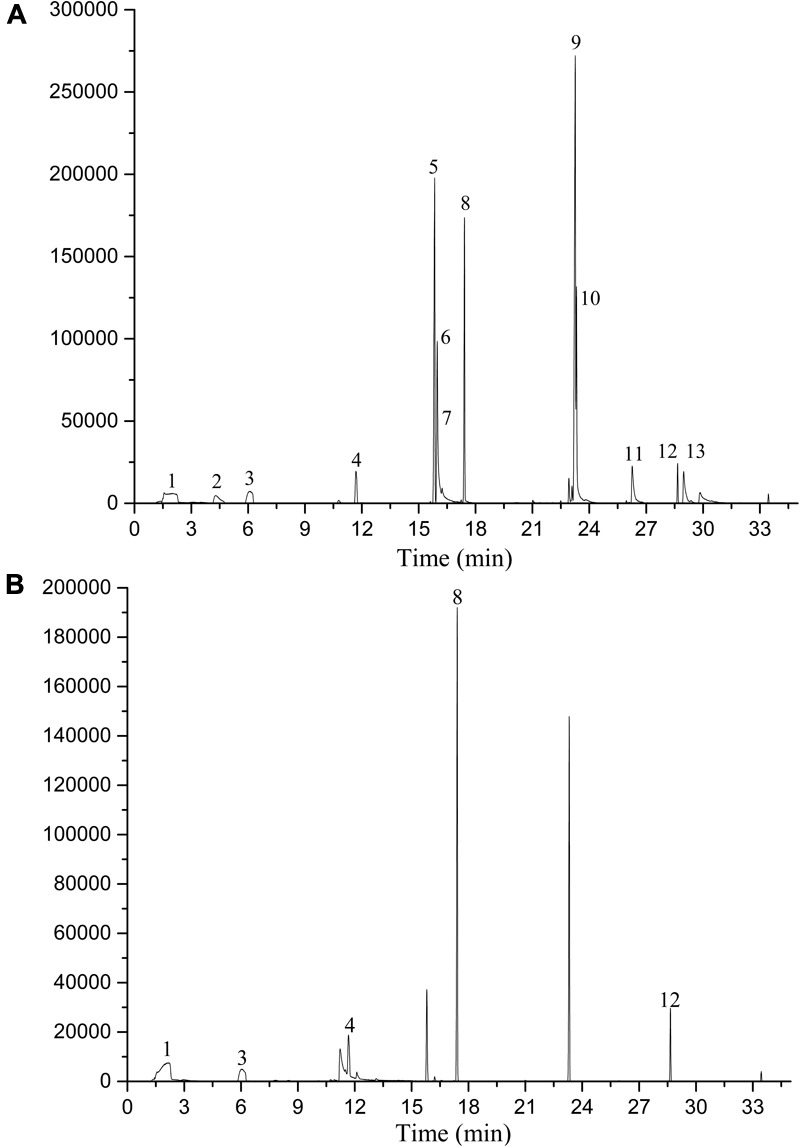
GC profiles of bacterial volatile analysis. The volatiles were extracted by SPME and analyzed by GC-MS. *P. putida* 1A00316 fermentation broth **(A)**; 2216E medium **(B)**.

According to GC-MS analysis (**Figure 5**), eight VOCs (peak area >1%) from strain 1A00316 fermentation broth accounted for 66.71% of the total area and were identified by SPME-GC/MS as dimethyl-disulfide, 1-undecene, 2-nonanone, 2-octanone, (Z)-hexen-1-ol acetate, 2-undecanone, 1-(ethenyloxy)-octadecane, and (Z)-3-decen-1-ol acetate (**Table 1**). Commercially available VOCs with similarity index >850 from the database search were chosen to test NA.

**Table 1:** GC/MS analysis of *P. putida* 1A00316 fermentation broth.

Compound	RT (min)	Relative (%)	Mw	Peak number
Dimethyl-disulfide	4.3677	1.2082	94.20	2
1-Undecene	15.8418	17.5697	154.29	5
2-Nonanone	15.9762	11.7213	142.24	6
2-Octanone	16.2363	1.0448	128.21	7
(Z)-Hexen-1-ol acetate	22.9257	1.2278	325.29	9
2-Undecanone	23.2595	26.8984	170.29	10
1-(Ethenyloxy)-octadecane	26.2596	3.5459	296.53	11
(Z)-3-decen-1-ol acetate	28.9821	3.4962	198.30	13

With the aim of exploring the potency of seven of these VOCs [(Z)-3-decen-1-ol acetate was not commercially available], their NA was tested on juveniles of *M. incognita in vitro*. 2-Octanone, (Z)-hexen-1-ol acetate, and 2-undecanone were the most active, showing LC_50_ values at 24 and 48 h of 23.714 and 22.712 mg/L, 33.922 and 32.351 mg/L, and 27.810 and 22.872 mg/L, respectively. The results of dimethyl-disulfide and 2-nonanone were 139.082 and 134.330 mg/L and 70.977 and 63.320 mg/L, respectively. 1-Undecene and 1-(ethenyloxy)-octadecane were not active at the tested concentration (**Table 2**). Interestingly, only 2-undecanone had fumigation activity against *M. incognita*, with an LC_50_ value at 48 h of 185.298 mg/L and an LC_90_ of 672.244 mg/L. Other identified VOCs had no fumigation activity against *M. incognita* even at 2000 mg/L (Supplementary Table S2).

**Table 2:** LC_50_ and FL values of commercial nematicidal compounds against *M. incognita*.

Compound	24 h		48 h	
	LC_50_ (mg/L)	FL (mg/L)	LC_50_ (mg/L)	FL (mg/L)
Dimethyl-disulfide	139.082	No	134.330	No
1-Undecene	>1000		>1000	
2-Nonanone	70.977	59.638–81.071	63.320	53.249–72.183
2-Octanone	23.714	1.134–39.489	22.712	19.852–25.280
(Z)-Hexen-1-ol acetate	33.922	28.875–39.180	32.351	28.021–36.826
2-Undecanone	27.810	24.887–31.022	22.872	20.078–25.678
1-(Ethenyloxy)-octadecane	>1000		>1000	
(Z)-3-decen-1-ol acetate	ND		ND	

*FL, fiduciary limits; ND, not determined.*

### Effect of Commercial VOCs on Egg Hatching of *M. incognita*

**FIGURE 6:**
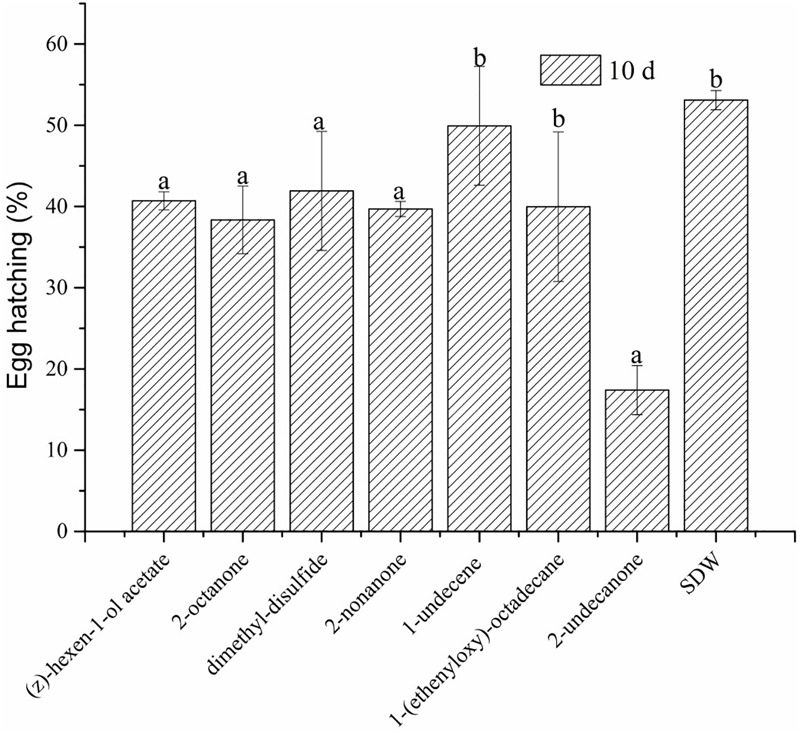
Effects of seven VOCs on egg hatching of *M. incognita* after 10 days in direct-contact (Z)-hexen-1-ol acetate, 2-octanone, dimethyl-disulfide and 2-nonanone at 200 mg/L, 1-undecene and 1-(ethenyloxy)-octadecane at 250 mg/L, 2-undecanone at 40 mg/L. Values with the same lowercase letters do not differ from each other at *P* < 0.05; bars indicate the standard error of the means (*n* = 3).

**FIGURE 7:**
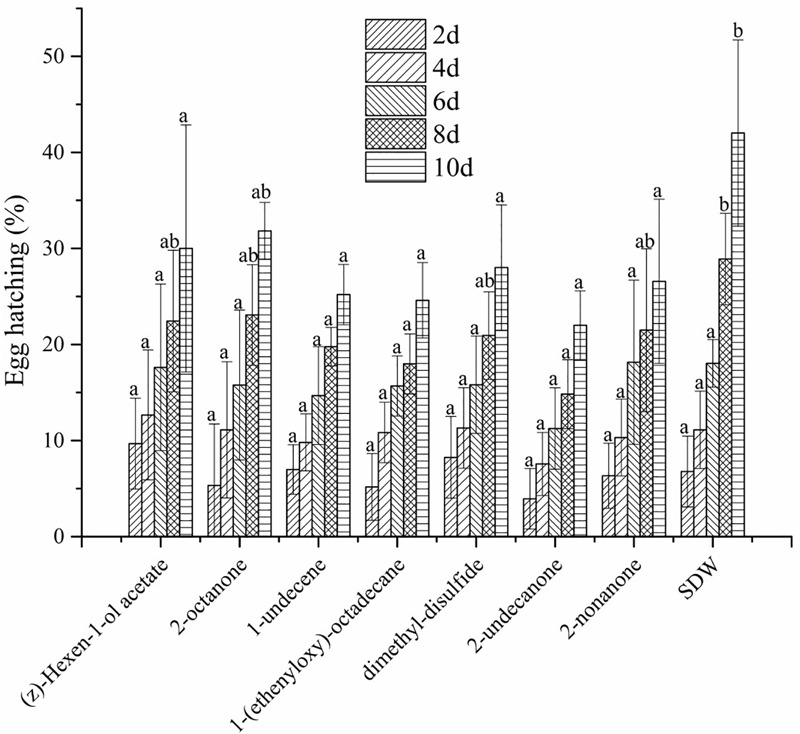
Fumigation by seven VOCs at 1000 mg/L on egg hatching of *M. incognita* after 2, 4, 6, 8, and 10 days of incubation. Values with the same lowercase letters do not differ from each other at *P* < 0.05; bars indicate the standard error of the means (*n* = 4).

The effect of the seven commercial organic compounds was measured *in vitro* by direct-contact. The results of the tested concentrations suggested that all seven VOCs had adverse effects on egg hatching of *M. incognita* (see Supplementary Tables S3–S9). (Z)-hexen-1-ol acetate at concentrations from 100 to 200 mg/L, 2-octanone at 200 mg/L, and dimethyl-disulfide and 2-nonanone from 50 to 200 mg/L slightly but significantly inhibited egg hatching, and 1-undecene at from 500 to 1000 mg/L and 1-(ethenyloxy)-octadecane at 1000 mg/L also inhibited egg hatching (Supplementary Tables S3–S9). Compared to SDW (**Figure 6**), (Z)-hexen-1-ol acetate, 2-octanone, dimethyl-disulfide, and 2-nonanone at 200 mg/L showed good inhibition of egg hatching, but 1-undecene and 1-(ethenyloxy)-octadecane at 200 mg/L showed no significant difference. Conversely, 2-undecanone at 40 mg/L was strongly inhibitory to egg hatching. We also determined whether the seven VOCs had volatile activity against egg hatching of *M. incognita*. Compared to SDW, the seven VOCs did not inhibit egg hatching after 2, 4, and 6 days of exposure, but egg hatching slowly declined after 8 days, consistent with slight ability to inhibit the egg hatching of *M. incognita* (**Figure 7**).

### Chemotaxis of Culture Filtrates and VOCs by J2 Juveniles of *M. incognita*

**FIGURE 8:**
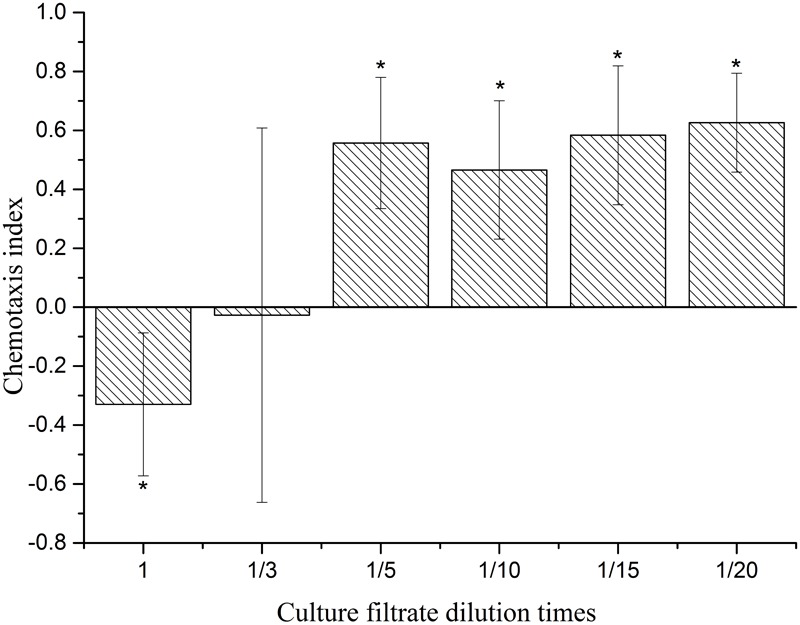
Chemotaxis of *M. incognita* J2 to 1A00316 culture filtrates. Values with the ^∗^ do not differ from each other at *P* < 0.05, they means the side of the number of attracting or avoiding nematodes is significantly different from another side; bars indicate the standard error of the means (*n* = 3).

Chemotaxis of culture filtrates from strain 1A00316 is presented in **Figure 8**. The results show that the higher concentrations of culture filtrates (original concentration and 1/3 original concentration) immobilized J2 nematodes (-1 < C.I. < 0) and acted as an attractant (0 < C.I. < 1) at from 1/5 to 1/20 original concentration. Interestingly, many nematodes were found in the test area (A), and the seven VOCs at concentrations of 1–10,000 mg/L showed C.I. ranging from -1 to 0 (**Table 3**). These results indicated that the seven VOCs repelled *M. incognita* J2 juveniles. Among them, the C.I. of different concentrations of (Z)-hexen-1-ol acetate, dimethyl-disulfide, and 2-nonanone showed no significant difference from the control. However, with the increase of concentration of 2-octanone, 1-undecene, 1-(ethenyloxy)-octadecane, and 2-undecanone, the absolute value of the C.I. gradually increased and the four VOCs had greater ability to repel nematodes. Comparing the C.I. of all seven VOCs at different concentrations, we found that 2-undecanone showed the greatest effect, with a C.I. value of -0.725 at 10,000 μg/mL.

**Table 3:** Chemotaxis index values of commercial VOCs toward *M. incognita.*

Compound	Chemotaxis index (C.I.)				
	1 mg/L	10 mg/L	100 mg/L	1000 mg/L	10,000 mg/L
(Z)-hexen-1-ol acetate	-0.42 ± 0.06a, B	-0.47 ± 0.05a, AB	-0.42 ± 0.13a, A	-0.53 ± 0.15a, A	-0.58 ± 0.20a, AB
2-Octanone	-0.41 ± 0.13a, B	-0.43 ± 0.08ab, AB	-0.44 ± 0.14ab, A	-0.55 ± 0.05ab, A	-0.56 ± 0.11b, AB
1-Undecene	-0.36 ± 0.15a, AB	-0.33 ± 0.12a, A	-0.32 ± 0.20a, A	-0.34 ± 0.09a, A	-0.56 ± 0.05b, AB
1-(Ethenyloxy)-octadecane	-0.23 ± 0.10a, A	-0.42 ± 0.12ab, AB	-0.45 ± 0.20b, A	-0.48 ± 0.16b, A	-0.49 ± 0.18b, A
Dimethyl-disulfide	-0.45 ± 0.10a, B	-0.43 ± 0.15a, AB	-0.48 ± 0.05a, A	-0.54 ± 0.07a, A	-0.54 ± 0.06a, A
2-Undecanone	-0.50 ± 0.08a, B	-0.53 ± 0.03a, B	-0.49 ± 0.15a, A	-0.54 ± 0.03a, A	-0.73 ± 0.12b, B
2-Nonanone	-0.42 ± 0.13a, B	-0.43 ± 0.11a, AB	-0.42 ± 0.15a, A	-0.46 ± 0.28a, A	-0.48 ± 0.10a, A

*Each value represents the average (±SE) of three replicates. Different lowercase letters indicate significant differences among different concentrations of the same VOC (LSD test, *P* < 0.05). Different uppercase letters indicate significant differences among different VOCs of the same concentration (LSD test, *P* < 0.05).*

## Discussion

In a preliminary experiment, different concentrations of culture filtrate from strain 1A00316 were added to 96- or 24-well tissue culture plates to test NA against eggs or J2 juveniles of *M. incognita*. This work showed effects greater than 90% killing of *M. incognita* J2 juveniles in the control group situated near culture filtrates, leading us to test SDW and culture filtrates in separate plates. The egg hatching in different 24-well plates containing 150 eggs, with SDW coexisting with different concentrations of culture filtrate in other wells, had significantly different inhibition effects on egg hatching after 10 days (Supplementary Figure S1). These results suggested that VOCs could contribute to the decrease in egg hatching in culture filtrates of strain 1A00316.

Based on the above experiment, we hypothesized that strain 1A00316 may produce volatiles to kill *M. incognita* J2 juveniles and inhibit egg hatching, and that there may be multiple modes of nematode control of *M. incognita.*

Subsequently, we identified eight VOCs with SPME-GC/MS analysis: dimethyl-disulfide, 2-nonanone, 2-undecanone, 2-octanone, 1-undecene, 1-(ethenyloxy)-octadecane, and (Z)-hexen-1-ol acetate. Dimethyl-disulfide, 2-nonanone, and 2-undecanone exhibited strong NA (NAs > 80%) against both juveniles and eggs at a concentration of 0.5 mmol by fumigation after 7 days of exposure (Huang et al., 2010). In another study, dimethyl-disulfide had the strongest NA (LC_90_ = 0.162 mmol/L) against *Bursaphelenchus xylophilus* exposed for 24 h in direct-contact (Yu et al., 2015). 2-Nonanone and 2-undecanone were reported to induce paralysis in *M. incognita* and *M. javanica* by direct-contact (Ntalli et al., 2011). However, to our knowledge, no NA has been reported for 2-octanone, 1-undecene, 1-(ethenyloxy)-octadecane, and (Z)-hexen-1-ol acetate against *M. incognita*. In our study, we found the strongest direct contact NA by dimethyl-disulfide, 2-nonanone, 2-octanone, (Z)-hexen-1-ol acetate, and 2-undecanone, but only 2-undecanone had fumigant activity against J2 juveniles. In addition, the seven VOCs had the ability to inhibit egg hatching of *M. incognita* both by direct-contact and as a fumigant.

Our further analysis focused on chemotaxis by J2 juveniles of *M. incognita*, and we found that they were repelled by higher concentrations of culture filtrate and attracted to lower concentrations. In recent years, there has been a proliferation of research on the chemotaxis of nematicidal VOCs. Hu et al. (2012) reported that *Chaetomium globosum* NK102 repelled *M. incognita* chemotaxis, but in that study the whole colony was regarded as a research object and the authors did not explore which factors acted as repellants of the nematode. In our study, all of the identified VOCs had a phobotactic effect on the nematodes, which explains the repellant activity of higher concentrations of the culture filtrates against *M. incognita.* In nature, VOCs can also exhibit an attractant effect: for example, the bacterium *Bacillus nematocida* B16 lures the nematode by emitting six potent VOCs, of which benzyl benzoate, benzaldehyde, 2-heptanone, and acetophenone were potent attractants, with the bacteria then entering the nematode intestine and causing death (Niu et al., 2010). These examples demonstrate the diversity and complexity of the VOCs against nematodes.

In summary, the results of our experiments using three-compartment Petri dishes and identification by GC/MS were consistent with the hypothesis that strain 1A00316 may produce VOCs with multiple modes of nematode control. We identified eight VOCs from strain 1A00316. Among them, dimethyl-disulfide, 2-nonanone, 2-octanone, (Z)-hexen-1-ol acetate, and 2-undecanone had strong NA in direct contact with *M. incognita* J2 juveniles, but only 2-undecanone had fumigant activity. In addition, the seven VOCs inhibited egg hatching both by direct-contact and as a fumigant. All of the seven VOCs repelled *M. incognita*. Our results showed that the VOCs from strain 1A00316 have at least three modes by which to control *M. incognita*: NA, fumigant activity, and repellent activity. The VOCs also acted on different stages in the nematode life cycle including J2 juveniles and eggs. The multiple modes of action of the VOCs produced from *P. putida* 1A00316 are consistent with the potential of the strain to be an effective biocontrol agent against *M. incognita* in the greenhouse. Further investigation is needed to understand the molecular mechanisms responsible for the NA of the VOC compounds produced by strain 1A00316.

## Author Contributions

YZ conceived and designed the work that led to the submission, acquired data, and played an important role in interpreting the results. ZS and GL provided the strain *Pseudomonas putida* 1A00316. MC and LZ provided the suggestions and helped to perform the analysis with constructive discussions. DH and WC helped to perform the analysis with constructive discussions. LT and DW drafted and revised the manuscript. ZY provided a platform for the experiments. JZ drafted and revised the manuscript and approved the final version.

## Conflict of Interest Statement

The authors declare that the research was conducted in the absence of any commercial or financial relationships that could be construed as a potential conflict of interest.
