# Supplementary material for: Multiple Modes of Nematode Control by Volatiles of Pseudomonas putida 1A00316 from Antarctic Soil against Meloidogyne incognita
Source: Front Microbiol. 2018 Feb 23;9:253. doi: 10.3389/fmicb.2018.00253 (PMC5863520; doi:10.3389/fmicb.2018.00253)
Supplement: Supplementary file 1 [file Data_Sheet_1.docx]

**Supplementary information**


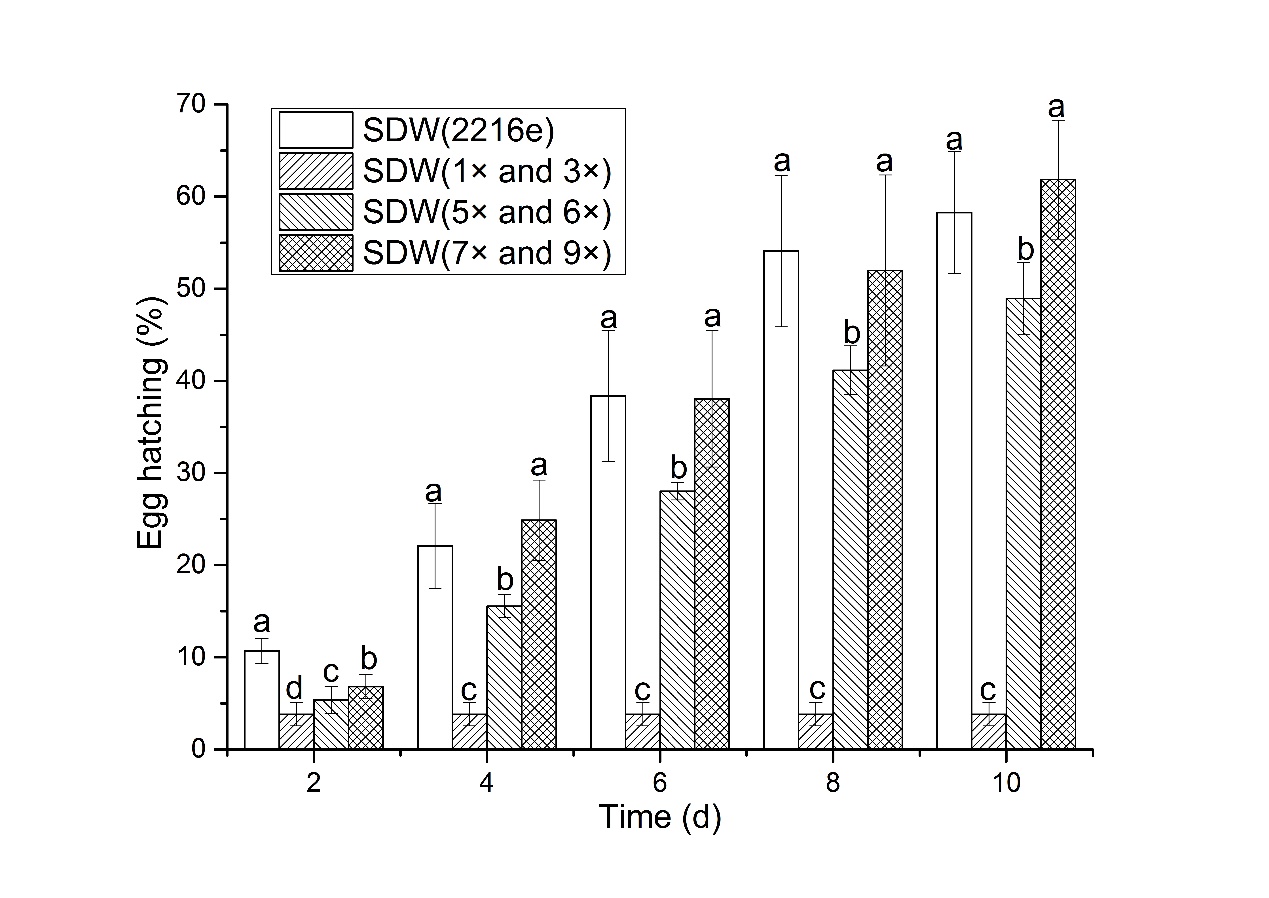


**Figure S1 Effects of SDW in 24-well plates on egg hatching of *M. incognita* at 2, 4, 6, 8 and 10 days after inoculation**. 200 μL SDW (sterile distilled water) was added to a well of a 24-well tissue culture plate, and other wells of the plate contained 2216e medium, full strength culture filtrate, or filtrate dilutions of 1/3, 1/5, 1/6, 1/7, and 1/9. Values with the same superscripts do not differ from each other at P˂0.05; bars indicate the standard error of the means (n= 3).

**Table S1 Nematicidal activity of culture filtrate VOCs of strain 1A00316 against *C. elegans* in a three-compartment Petri plate**

| Time (h) | Mortality (%) | |
| --- | --- | --- |
|  | *C. elegans* | CK |
| 24 | 2.64±0.58 | 2.83±1.13 |
| 48 | 6.07±2.13 | 3.92±1.22 |
| 72 | 8.28±2.56 | 5.92±1.21 |

| **Table S2 Fumigant effects of seven commercial VOCs against *M. incognita* J2 juveniles**Compound | 48 h | | | | |
| --- | --- | --- | --- | --- | --- |
|  | LC_50_ (mg/L) | FL (mg/L) | LC_50_ (mg/L) | | FL (mg/L) |
| Dimethyl-disulfide | — | — | — | | — |
| 1-Undecene | — | — | — | | — |
| 2-Nonanone | — | — | — | | — |
| 2-Octanone | — | — | — | | — |
| (Z)-Hexen-1-ol acetate | — | — | | — | — |
| 2-Undecanone | 185.298 | 162.378-201.427 | | 672.244 | 519.781-733.794 |
| 1-(Ethenyloxy)-octadecane | — | — | | — | — |
| (Z)-3-Decen-1-ol acetate | ND |  | | ND |  |

FL=Fiduciary Limits, ND=Not determined, “—” no activity.

**Table S3 Effect of (Z)-hexen-1-ol-acetate on egg hatching of *M. incognita* by direct-contact.** Within each treatment time, bars designated with the same letter are not significantly different at the 0.05 level.

| Time | Egg hatching (%) | | | |
| --- | --- | --- | --- | --- |
|  | 200 mg/L | 100 mg/L | 50 mg/L | SDW |
| 2d | 6.75±2.50a | 9.29±3.84a | 9.95±4.81a | 11.88±4.21a |
| 4d | 13.11±3.65a | 15.13±5.47a | 14.16±5.47a | 19.39±2.47a |
| 6d | 20.37±3.21a | 21.71±3.99a | 19.44±5.59a | 26.48±3.65a |
| 8d | 28.71±2.40a | 25.98±4.40a | 28.00±9.99a | 34.11±1.87a |
| 10d | 40.70±1.11a | 40.16±4.03a | 45.46±8.02a | 53.10±1.18b |

**Table S4 Effect of 2-octanone on egg hatching of *M. incognita* by direct-contact*.*** Within each treatment time, bars designated with the same letter are not significantly different at the 0.05 level.

| Time | Egg hatching (%) | | |
| --- | --- | --- | --- |
|  | 200 mg/L | 50 mg/L | SDW |
| 2d | 6.23±2.80a | 10.70±0.93a | 11.88±4.21a |
| 4d | 13.73±1.32a | 14.46±1.68a | 19.39±2.47b |
| 6d | 17.49±2.65a | 24.25±1.30b | 26.48±3.65b |
| 8d | 24.19±4.75a | 31.93±2.80b | 34.11±1.87b |
| 10d | 38.34±4.16a | 47.36±6.03b | 53.10±1.18b |

**Table S5 Effect of dimethyl-disulfide on egg hatching of *M. incognita* by direct-contact*.*** Within each treatment time, bars designated with the same letter are not significantly different at the 0.05 level.

| Time | Egg hatching (%) | | |
| --- | --- | --- | --- |
|  | 200 mg/L | 50 mg/L | SDW |
| 2d | 7.20±2.08a | 6.64±3.38a | 11.88±4.21a |
| 4d | 11.95±2.48a | 13.54±1.81a | 19.39±2.47b |
| 6d | 22.99±2.61a | 21.77±2.81a | 26.48±3.65a |
| 8d | 27.37±5.01a | 29.50±2.44a | 34.11±1.87a |
| 10d | 41.92±7.34a | 41.45±3.37a | 53.10±1.18b |

**Table S6 Effect of 2-nonanone on egg hatching of *M. incognita* by direct-contact*.*** Within each treatment time, bars designated with the same letter are not significantly different at the 0.05 level.

| Time | Egg hatching (%) | | |
| --- | --- | --- | --- |
|  | 200 mg/L | 50 mg/L | SDW |
| 2d | 9.49±3.49a | 7.95±2.65a | 11.88±4.21a |
| 4d | 11.80±3.44a | 12.20±3.81a | 19.39±2.47b |
| 6d | 23.33±4.54a | 22.19±3.28a | 26.48±3.65a |
| 8d | 28.87±3.18a | 29.68±7.94a | 34.11±1.87a |
| 10d | 39.69±0.93a | 41.98±5.16a | 53.10±1.18b |

**Table S7 Effect of 1-undecene on egg hatching of *M. incognita* by direct-contact*.*** Within each treatment time, bars designated with the same letter are not significantly different at the 0.05 level.

| Time | Egg hatching (%) | | | |
| --- | --- | --- | --- | --- |
|  | 1000 mg/L | 500 mg/L | 250 mg/L | SDW |
| 2d | 6.64±2.80a | 8.13±2.84a | 13.16±6.41a | 11.88±4.21a |
| 4d | 11.30±3.37a | 13.80±2.25a | 19.20±2.84b | 19.39±2.47b |
| 6d | 19.16±4.15a | 16.78±2.07a | 29.37±1.69b | 26.48±3.65b |
| 8d | 25.38±9.44a | 21.93±10.94a | 33.90±1.39a | 34.11±1.87a |
| 10d | 27.79±11.34a | 32.77±9.34a | 49.93±7.31b | 53.10±1.18b |

**Table S8 Effect of 1-(ethenyloxy)-octadecane on egg hatching of *M. incognita* by direct-contact*.*** Within each treatment time, bars designated with the same letter are not significantly different at the 0.05 level.

| Time | Egg hatching (%) | | |
| --- | --- | --- | --- |
|  | 1000 mg/L | 250 mg/L | SDW |
| 2d | 5.86±1.61a | 15.24±5.95b | 11.88±4.21a |
| 4d | 9.51±1.89a | 17.99±5.57b | 19.39±2.47b |
| 6d | 12.87±1.56a | 21.47±4.34b | 26.48±3.65b |
| 8d | 17.91±5.57a | 29.27±6.43b | 34.11±1.87b |
| 10d | 22.32±7.16a | 39.97±9.21b | 53.10±1.18b |

**Table S9 Effect of 2-undecanone on egg hatching of *M. incognita* by direct-contact*.*** Within each treatment time, bars designated with the same letter are not significantly different at the 0.05 level.

| Time | Egg hatching (%) | | | |
| --- | --- | --- | --- | --- |
|  | 40 mg/L | 30 mg/L | 20 mg/L | SDW |
| 2d | 5.60±1.33a | 9.42±4.79a | 6.81±2.85a | 11.88±4.21a |
| 4d | 5.87±0.86a | 14.08±4.16b | 14.76±2.65b | 19.39±2.47b |
| 6d | 9.86±0.74a | 21.74±4.37b | 21.60±6.11b | 26.48±3.65b |
| 8d | 12.67±2.03a | 29.72±11.14b | 28.12±6.15b | 34.11±1.87b |
| 10d | 17.40±3.02a | 42.80±8.05b | 40.06±8.12b | 53.10±1.18bc |
